# Honesty Versus Hypocrisy

**Published:** 1885-02

**Authors:** 


### HONESTY VERSUS HYPOCRISY.

The *New York Medical Times* is an able, honest journal. When its editors gave up Homœopathy they sensibly and honestly dropped the name. Now these able gentlemen would have the American Institute do likewise. Having ceased to represent Homœopathy, they should also cease to bear its name. The *Times*, in its January issue, says:

"One has only to look over the volume containing the Transactions of the Society, * * * * to be convinced of the appropriateness of the change. Nine-tenths of the articles in this volume * * * * might just as well have been read before an old school or an eclectic association as one bearing a homœopathic name. In fact, there is scarcely anything in the book, so far as the papers are concerned, which would mark this volume as the transactions of a homœopathic society."

If this be true, one may well ask: Why does the Institute exist?

Well may Dr. Wood exclaim: "Little by little is creeping out that which the regular profession has long known, namely, that for a man to be a homœopathic physician at present necessitates that he be ignorant, foolish, or knavish—that is, if it be knavish 'to live a lie.'"

